# Acute Onset of Delayed Facial Nerve Paralysis After an Uncomplicated Total Parotidectomy for an Oncocytoma

**DOI:** 10.7759/cureus.55347

**Published:** 2024-03-01

**Authors:** Tyler J Ostrowski, Richa S Nathan, Luke Mammen, Neil Gildener-Leapman

**Affiliations:** 1 Otolaryngology - Head and Neck Surgery, Albany Medical Center, Albany, USA; 2 Otolaryngology - Head and Neck Surgery, Albany Medical College, Albany, USA

**Keywords:** steroid use, head and neck pathologies, total parotidectomy, parotid oncocytoma, delayed facial nerve palsy

## Abstract

Facial nerve injury is one of the most substantial potential sequelae of parotid surgery. Pulling, stretching, and otherwise disturbing the facial nerve during parotid surgery can lead to post-surgical neural paresis and consequential deficits in facial movement. Furthermore, transection of the main facial nerve trunk or its branches, either purposeful or incidental, can lead to complete paralysis of the related facial musculature. Facial nerve injury is often diagnosed immediately post-operatively as evident by deficits in ipsilateral facial motion on examination of the patient in the recovery unit or, at most, by one week post-operatively. Although delayed onset facial nerve paralysis is seen in traumatic injury and otologic surgery, it is uncommon that facial nerve paralysis presents late after parotid surgery in the absence of hematoma development, viral reactivation, or secondary insult. Here, we present the case of a 70-year-old man developing a delayed acute onset of hemi-facial paralysis 12 days after right-sided total parotidectomy for an oncocytoma; a facial nerve examination done immediately post-operatively and at the one-week post-operative follow-up was found to be normal. The patient was treated with two courses of high-dose oral steroids with close-to-complete resolution.

## Introduction

Approximately 3%-10% of tumors in the head and neck region occur in the salivary glands. Of these, parotid gland tumors make up 80% of all salivary gland tumors [1]. Oncocytomas are rare epithelial cell-derived tumors that generally occur in the salivary tissue in patients over the age of 50. Although most salivary tumors are benign in nature, many require surgical intervention. Facial nerve palsy is a commonly feared complication following parotidectomy, even for benign tumors. Despite advances in surgical techniques, facial nerve paresis and paralysis are common sequela of parotid surgery due to the intimate relationship of the facial nerve with the parotid gland. Up to 64% of patients experience temporary facial nerve weakness and up to 9% experience permanent facial nerve weakness following parotidectomy [2]. Most temporary facial nerve palsies following parotidectomy arise during post-operative days 1-5 and resolve within six months, with approximately 90% of deficits resolving within one month. In 1985, House and Brackmann presented a six-point grading scale for facial nerve function at the American Academy of Otolaryngology - Head and Neck Surgery Annual Meeting as a means of standardizing how we describe facial nerve function. Although numerous grading systems exist to document facial nerve function, the ease and practicality of the House-Brackmann scale became the primary scale used to define facial nerve function by physical examination [3-5]. The House-Brackmann grading system defines the degree of neural dysfunction on a scale of I to VI, with Grade I representing normal function in all areas of the face and Grade VI defining total paralysis with no facial movement. In brief, a House-Brackmann score of II represents slight facial weakness with normal facial tone and symmetry, complete eye closure without effort, and a slight mouth weakness only noticeable on close inspection. Grade III defines patients with hardly noticeable asymmetry at rest, if at all, and moderate dynamic dysfunction, along with obvious but not disfiguring facial asymmetry, as with full effort effort they are able to achieve moderate mouth and forehead movement and full eye closure. Grade IV defines severe dysfunction consisting of obvious facial weakness, incomplete eye closure, no forehead movement, and asymmetric movement of the mouth. Grade V is defined as little ability to make any facial expressions, no forehead movement, and incomplete eye closure [4].

The incidence of facial nerve palsy is found to be higher with a total parotidectomy as compared to superficial; this is likely due to a stretch injury of the facial nerve [1]. Facial nerve dysfunction rarely develops more than one week after surgery. Facial nerve palsies are a substantial risk associated with parotid surgery but are not unique to this procedure. For example, facial nerve paralysis can occur during transmastoid surgical approaches requiring manipulation of structures surrounding the facial recess and the facial canal in the petrous aspect of the temporal bone [2]. Facial nerve palsies are also seen because of viral infection of the facial nerve, trauma to the temporal bone or facial soft tissue, and inner ear surgical complications.

## Case presentation

A 70-year-old male presented to our head and neck surgery clinic in April 2023 after being referred from a community otolaryngologist for the evaluation of a parotid mass. Prior to the presentation, the patient had undergone neck soft tissue CT that showed a right-sided, bilobed parotid tumor with slightly irregular borders involving the deep lobe of the parotid gland. He had also had a nondiagnostic ultrasound-guided biopsy at that time. The patient did disclose that he had felt occasional mild, right-sided facial pain overlying the site of the mass. He denied any facial weakness or numbness. He also denied nasal obstruction, changes in hearing, otalgia, dysphagia, odynophagia, or unintended weight loss. The patient denied using tobacco products and endorsed social alcohol use. The patient’s physical examination at this time was unremarkable, including a normal neck exam with no signs of lymphadenopathy, normal parotid and submandibular glands and ducts bilaterally, an absence of cutaneous skin lesions in the head or neck, and a normal cranial nerve II-XII function on examination. The point-of-care ultrasound at this visit was unable to adequately demonstrate the right parotid mass due to the depth.

The patient underwent a right total parotidectomy with cranial nerve VII dissection and preservation in June 2023. Nerve monitors in the right orbicularis oculi and orbicularis oris muscles were confirmed to be adequately functioning prior to the start of the surgery. After identifying the standard facial nerve landmarks such as the tragal pointer and posterior belly of the digastric muscle, the facial nerve was isolated using blunt dissection and a harmonic device. The main trunk of the facial nerve was intimately involved with the right parotid mass, and extensive dissection, including retrograde dissection from the marginal mandibular nerve, was necessary to safely remove the lobulated tumor. The main trunk of the facial nerve was stimulated and demonstrated to be intact with 0.4-mA stimulation showing good response in both inferior and superior divisions at the completion of the surgery. A surgical closed system drain was placed and closure of the wound was performed in a standard manner. The patient returned to the post-anesthesia care unit after extubation in a stable condition. The patient had a normal cranial nerve VII function (House-Brackmann Grade I) bilaterally in the immediate postoperative period. Final surgical pathology of the tumor showed a completely excised, non-malignant, 2.5-cm right-sided oncocytoma.

The patient followed up in the clinic on post-operative day 3 for drain removal. At this visit, the surgical site was healing as expected with an incision that was clean, dry, and intact, and no significant or unexpected ecchymosis or swelling. The patient had a House-Brackmann Grade I function bilaterally at this time. The patient returned to the clinic on post-operative day 10 for suture removal, at which point the patient had continued to have an uncomplicated post-operative course. He noted mild right-sided ear pressure and pre-auricular numbness but denied any facial weakness. His incision remained clean, dry, and intact with no significant or unexpected ecchymosis or swelling. The House-Brackmann score remained Grade I at this visit. A standard follow-up was scheduled in four months.

On post-operative day 17, the patient called the clinic regarding acute onset, right-sided facial nerve weakness that arose on post-operative day 12. Symptoms included increased right eye tearing and difficulty with eyelid closure accompanied by difficulty closing his mouth leading to liquid leaking out upon drinking. He denied any surgical site ecchymosis, swelling, or external insult. The patient also denied any neurologic symptoms such as confusion or mental status change at this time. From a review of the history and photographs, it appeared the patient was experiencing delayed facial nerve paralysis, likely due to an immune or inflammatory etiology, in light of his normal postoperative findings and an otherwise normal physical examination. The patient did relay that his daughter had a remote history of idiopathic facial nerve paralysis as an adult, which independently resolved.

**Figure 1:**
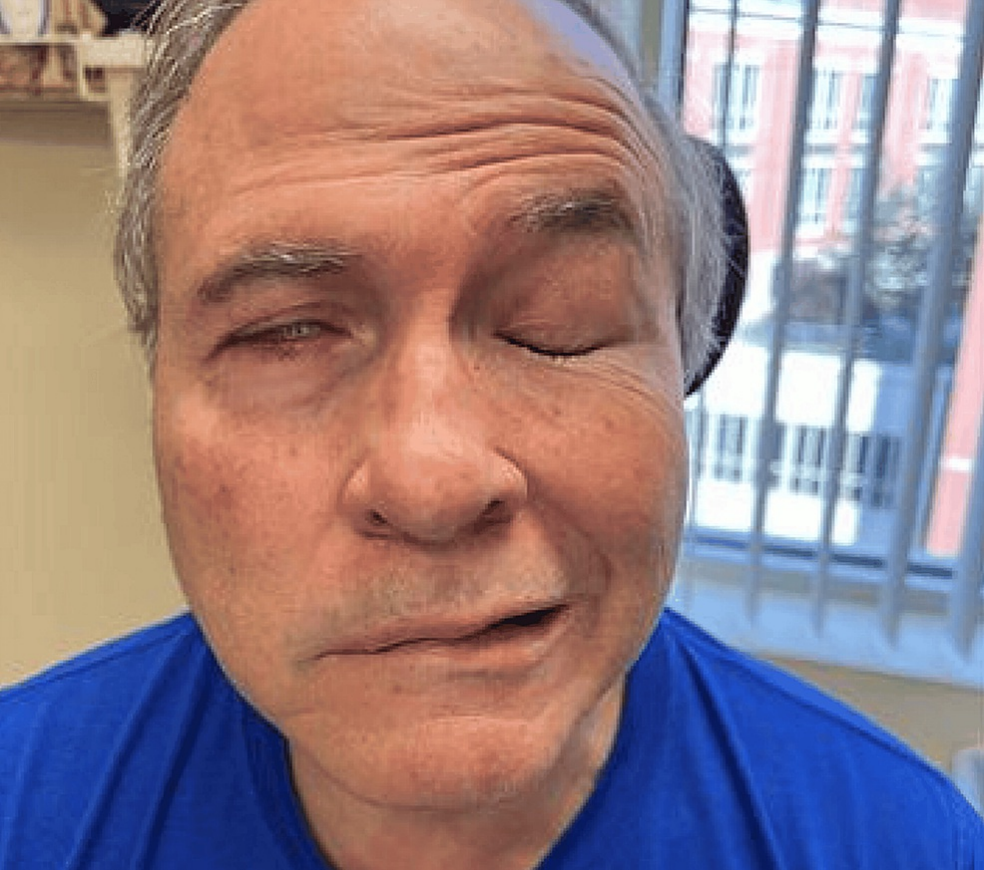
Postoperative day 19 clinic visit showing the patient attempting to smile and close his eyes tight. Notice the right-sided mouth droop, incomplete eye closure, and minimal forehead movement.

The patient was urgently seen in the clinic (Figure 1). He demonstrated 6/6 facial nerve paralysis on the right side. Due to the perceived inflammatory or immune etiology of nerve dysfunction, the patient was prescribed a prednisone taper to be taken as follows: 20 mg orally three times a day for seven days, 20 mg orally twice a day for one day, 20 mg orally for one day, and 10 mg orally for one day. A lubricating eye ointment to use at bedtime was also prescribed at this visit.

The patient was called one week after starting the high-dose steroid taper; he reported mild improvement in his symptoms albeit still unable to completely close eyes. Due to the paucity of literature surrounding this case and the perceived improvement in symptoms, the high-dose steroid taper was renewed for a complete second course.

**Figure 2:**
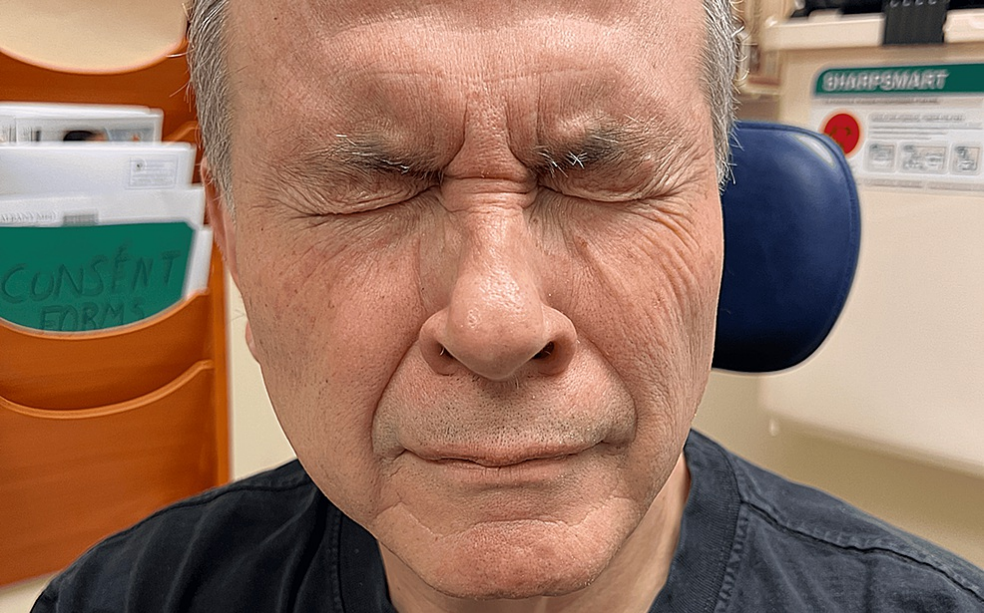
The patient seen at his 10-week postoperative clinic visit with improved right-sided facial strength and symmetry specifically with tight eye closure bilaterally.

The patient returned to clinic 10 weeks postoperatively with no complaints and a House-Brackmann grade II function on the right as demonstrated in Figure 2. The surgical site was healing as expected. The patient was instructed to follow up as needed.

## Discussion

The majority of salivary gland tumors occur in the parotid glands; although most of the tumors occurring in this gland are benign, surgery, namely, parotidectomy, is commonly recommended. Surgery is the gold standard of treatment for multiple reasons including varying sensitivities and specificities of diagnostic exams depending on tumor type, and the potential for tumor malignant transformation in some cases. The parotid gland is a bilaterally paired gland that consists of a superficial and deep lobe. The lobes of the gland are separated by the facial nerve and the posterior facial vein as they course within the glandular tissue [6]. Due to the intimate relationship of the nerve and the parotid gland, a meticulous dissection of the facial nerve off of the glandular tissue is imperative and much care must be taken to not violate the neural tissue. Although there are several reliable anatomical landmarks for identifying the facial nerve, such as the tragal pointer, posterior belly of the digastric muscle, and tympanomastoid suture, among others, facial nerve monitoring is widely used as an adjunctive safety measure because of the devastation associated with insult or injury to the facial nerve [7]. Although the literature varies, the rate of transient facial nerve dysfunction associated with parotidectomy with facial nerve dissection has been reported to be as high as 6.5% to 100%, and for permanent or long-term dysfunction, from 0.9% to 50.0% in [2,7].

This report presents the case of a 70-year-old male with a right-sided oncocytoma who developed acute right-sided facial nerve paralysis (House-Brackmann VI) 12 days after a total right-sided parotidectomy. The patient was then placed on a high-dose steroid taper for a presumed inflammatory etiology in the setting of an uncomplicated procedure and absence of surgical site abnormalities. Due to a paucity of evidence in the head and neck literature regarding delayed facial nerve paralysis, a second course of high-dose steroids was prescribed (by patient's request), which led to the resolution to a Grade II House-Brackmann function. Typically, in the postoperative period, the evaluation of facial nerve paralysis would involve concern for injury to the neural tissue during surgery recalled by the surgeon and documented in the operative summary, development of surgical site infection and concern for phlegmon or abscess development, and concern for the development of a postoperative hematoma, with the latter two causing mass effect on the nerve. Additionally, in these cases there should be a high suspicion for viral reactivation, specifically of herpes simplex virus-1 (HSV-1) that is known to cause facial nerve paralysis, especially in times of physical stressors such as surgery. As reported, our patient did not have a significant injury to his facial nerve during the procedure, and had almost two weeks of normal function postoperatively ruling out tissue insult as a cause of the dysfunction. Additionally, our patient had no signs of surgical site infection, phlegmon, abscess, or hematoma development. Finally, our patient denied a diagnosis of active or latent HSV-1 as well as a history of facial nerve paralysis in his past. The acute nature of facial nerve weakness at almost two weeks post-parotidectomy is extremely rare, particularly in the absence of the aforementioned etiologies. This has seldom appeared in the literature to date, as most cases are documented intraoperatively or immediately following surgery, particularly in days 1-5 [3].

Although rare, delayed facial nerve paralysis has been documented by several case reports published in the neurotology literature. From 1987 to 1996, of 1800 middle ear operations, there were 7 cases documented of ipsilateral, delayed facial nerve palsies [8]. Delayed facial nerve palsies are generally caused by anesthetic use, trauma, or occur due to iatrogenic causes in surgery, but viral reactivation, namely, HSV-1, inflammatory reactions to surgery or injury, and other less common etiologies can also be the reasons. Generally, the facial nerve sequela occurs within the first 72 hours after the insult, particularly in post-surgical cases. Multiple studies have cited delayed facial nerve palsies in the postoperative context of middle ear surgeries, most often attributed to HSV-1 reactivation. For example, a study by De Stefano et al. discusses a case of facial nerve paralysis arising on postoperative day 11 after a middle ear surgery due to HSV-1 infection and reactivation [9]. Another report published by Bonkowsky et al. discusses delayed facial nerve palsy arising within the first week after an uncomplicated middle ear surgery with an unidentified etiology [10]. Along the same lines, Shea discusses the incidence of facial nerve paralysis following stapes surgery in about 1 in every 1000 cases and suspects an immune etiology. Zohar and Laurian have added to this notion by reporting a case of delayed facial nerve paralysis after uncomplicated stapes surgery that was treated successfully to improvement in the neural hypofunction with prednisone [10-12]. In all of these cases, it was predicated that the rare occurrence of delayed facial nerve paralysis had a viral etiology.

Due to the course of our patient’s symptoms and based on the available data, it was presumed that the paralysis was secondary to an inflammatory reaction; thus, the patient was treated with a course of an oral steroid taper. Corticosteroids exert potent anti-inflammatory effects by altering several intracellular signal transduction pathways that normally encourage local cellular recruitment and subsequent tissue inflammation [13]. Although their efficacy is not clearly delineated, patients with unilateral facial paralysis due to Bell’s palsy (an acute peripheral facial palsy of an unknown cause) or paralysis secondary to traumatic, and iatrogenic sources, are often treated empirically with corticosteroids due to their anti-inflammatory properties [14]. One such study demonstrating the benefits of corticosteroid therapy in relation to Bell’s palsy showed that at the three-month follow-up, 83% of patients treated with prednisolone regained facial function opposed to 64% in the control group. Some difference was maintained at the nine-month follow-up timepoint with 94% of the prednisolone group compared to only 82% of the placebo group regaining function [14,15].

Due to the paucity of data directly related to the delayed onset of facial nerve paralysis in head and neck cases, particularly parotidectomy, our patient was treated with prednisone in accordance with the presumed etiology of our patient’s neural deficit being an inflammatory reaction. Our case followed the same pattern of the few similar documented cases in the otologic literature, showing improvement of symptoms after one course, and total recovery of the facial nerve function after two courses of a prednisone taper.

## Conclusions

Facial nerve paralysis is a substantial risk associated with parotidectomy due to the integral relationship of the facial nerve and the parotid gland. Injury to the facial nerve is not unique after parotid surgery; however, paresis or paralysis of the nerve generally reveals itself immediately postoperatively or within one week. We have presented a case of delayed facial nerve paralysis after an uncomplicated parotidectomy for an oncocytoma characterized by a delayed onset of facial nerve paralysis. In times of no surgical insult or injury to the nerve, and in the absence of hematoma, abscess, or other surgical site abnormalities, neural hypofunction is generally due to inflammatory responses. Although delayed paralysis has been documented in the otologic literature, this case is unique in that it is the first, to our knowledge, to describe the development and treatment of delayed facial nerve paralysis related to an uncomplicated parotid surgery with no signs of neural insult intraoperatively or in the immediate postoperative period. This case illustrates the good prognosis for neural recovery as seen in other settings of delayed paralysis of anatomically intact nerves.

## References

[1] Marchese-Ragona R, De Filippis C, Marioni G, Staffieri A (2005). Treatment of complications of parotid gland surgery. Acta Otorhinolaryngol Ital.

[2] Salih AM, Baba HO, Saeed YA (2022). Pattern of facial nerve palsy during parotidectomy: a single-center experience. J Int Med Res.

[3] Jin H, Kim BY, Kim H (2019). Incidence of postoperative facial weakness in parotid tumor surgery: a tumor subsite analysis of 794 parotidectomies. BMC Surg.

[4] House JW, Brackmann DE (1985). Facial nerve grading system. Otolaryngol Head Neck Surg.

[5] Reitzen SD, Babb JS, Lalwani AK (2009). Significance and reliability of the House-Brackmann grading system for regional facial nerve function. Otolaryngol Head Neck Surg.

[6] Bialek EJ, Jakubowski W, Zajkowski P, Szopinski KT, Osmolski A (2006). US of the major salivary glands: anatomy and spatial relationships, pathologic conditions, and pitfalls. Radiographics.

[7] Savvas E, Hillmann S, Weiss D, Koopmann M, Rudack C, Alberty J (2016). Association between facial nerve monitoring with postoperative facial paralysis in parotidectomy. JAMA Otolaryngol Head Neck Surg.

[8] Esaki S, Yamano K, Katsumi S, Minakata T, Murakami S (2015). Facial nerve palsy after reactivation of herpes simplex virus type 1 in diabetic mice. Laryngoscope.

[9] De Stefano A, Neri G, Kulamarva G (2009). Delayed facial nerve paralysis post middle ear surgery: herpes simplex virus activation. B-ENT.

[10] Bonkowsky V, Kochanowski B, Strutz J, Pere P, Hosemann W, Arnold W (1998). Delayed facial palsy following uneventful middle ear surgery: a herpes simplex virus type 1 reactivation?. Ann Otol Rhinol Laryngol.

[11] Shea JJ (1988). Thirty years of stapes surgery. J Laryngol Otol.

[12] Zohar Y, Laurian N (1985). Facial palsy following stapedectomy: a case report. J Laryngol Otol.

[13] Barnes PJ (2006). How corticosteroids control inflammation: Quintiles Prize Lecture 2005. Br J Pharmacol.

[14] Finsterer J (2008). Management of peripheral facial nerve palsy. Eur Arch Otorhinolaryngol.

[15] Sullivan FM, Swan IR, Donnan PT (2007). Early treatment with prednisolone or acyclovir in Bell's palsy. N Engl J Med.

